# Supplementary material for: Prevalence of corneal findings and their interrelation with hematological findings in monoclonal gammopathy
Source: PLoS One. 2022 Oct 31;17(10):e0276048. doi: 10.1371/journal.pone.0276048 (PMC9621422; doi:10.1371/journal.pone.0276048)
Supplement: S1 Protocol — (DOC) [file pone.0276048.s002.doc]

**Inzidenz der paraproteinämischer Keratopathie bei Patienten mit monoklonaler Gammopathie unklarer Signifikanz, smoldering multiplem Myelom und multiplem Myelom.**

**(Incidence of paraproteinemic keratopathie in patients with monoklonal gammopathie of undetermined significance, smoldering multiple myeloma und multiple myeloma.)**

*In der finalen Version wurde die Prävalenz anstatt der Inzidenz analysiert.*

**Sponsor:**

Augenklinik und Poliklinik, Universitätsmedizin Mainz, Johannes Gutenberg-Universität Mainz

**Studienleiterin:**

Dr. med. Joanna Wasielica-Poslednik

Oberärztin der Klinik

Adresse: Augenklinik und Poliklinik, Langenbeckstr. 1, 55131 Mainz

Telefon +49 6131 176038

E-Mail: joanna.wasielica-poslednik@unimedizin-mainz.de

**Synopsis**

| **Titel** | **Inzidenz der paraproteinämischen Keratopathie bei Patienten mit monoklonaler Gammopathie unklarer Signifikanz (MGUS), smoldering multiplem Myelom (SMM) und multiplem Myelom (MM).** |
| --- | --- |
| **Kurztitel** | **Paraproteinämische Keratopathie** |
| **Protokoll Nr.** | MZ-MGUS-2016 |
| **Sponsor** | Augenklinik und Poliklinik, Universitätsmedizin Mainz, Johannes Gutenberg-Universität Mainz |
| **Studiendesign** | Prospektive Kohortenstudie |
| **Studienpopulation** | Konsekutive Patienten, die innerhalb von 12 Monaten mit folgenden Diagnosen in der II Med. Klinik vorstellig werden:  **Gruppe 1**: Patienten mit MGUS  **Gruppe 2**: Patienten mit SMM  **Gruppe 3**: Patienten mit MM |
| **Einschlusskriterien** | **Gruppe 1**: **Patienten mit MGUS**  -Patienten mit monoklonaler Gammopathie im Urin und/oder Serum  - Kein Hinweis auf einen Endorgangschaden analog der IMWG-Kriterien (Niereninsuffzienz, Anämie, Knochenläsion, Hyperkalzämie)  - Plasmazellen <10% im Knochenmark und Paraprotein im Serum <30g/l und Paraprotein im Urin <500mg/24h  **Gruppe 2**: **Patienten mit SMM**   - Patienten mit monoklonaler Gammopathie im   Urin und/oder Serum   - Mehr als 10% Plasmazellen im Knochenmark   und/ oder Paraprotein von >30g/l im Serum und/oder Paraprotein im Urin >500mg/24h   - Kein Hinweis auf einen Endorgangschaden analog der IMWG-Kriterien (Niereninsuffzienz, Anämie, Knochenläsion, Hyperkalzämie) - Keine positiven Biomarker-Kriterien   **Gruppe 3**: **Patienten mit behandlungspflichtigem MM**   - siehe SMM+ - Entweder CRAB+:   -Hyperkalzämie: Serumkalzium gesamt >2,75mmol/l  - Anämie: Hb <10g/dl absolut, oder >2,5g/dl Differenz zum unteren Normbereich  -Mehr als eine osteolytische Knochenläsion  -Niereninsuffzienz: Kreatini-Clearance <=40ml/min und/oder Serumkreatinin >2mg/dl  - Und/oder Biomarker-Kriterien+:  -FLC-Ratio involved/uninvolved Leichtkette: >100  - Mehr als eine fokale Läsion im Ganzkörper MRT  - Klonale Plasmazellen >60% im Knochenmark  **Alle Gruppen:**   - Männer und Frauen - Alter ≥ 18 Jahre - Patient ist in der Lage, am Studienablauf teilzunehmen - Patient hat zur Studienteilnahme eingewilligt |
| **Ausschlusskriterien** | - Zustand nach beidseitigem refraktivem Hornhaut-Eingriff (z.B. LASIK, LASEK, PTK) - Ausschluss Patienten mit asekretorischem multiplen Myelom - Andere Hämatologische Grunderkrankung außer der MGUS/SMM/MM die mit dem Vorliegen einer monoklonale Gammopathie assoziiert ist |
| **Fragestellungen** | - Wie ist die Inzidenz der paraproteinämischer Keratopathie bei Patienten mit MGUS, SMM und MM?   *In der finalen Version wurde die Prävalenz anstatt der Inzidenz analysiert*   - Ist die Häufigkeit und der Trübungsmuster unterschiedlich in den o.g. Gruppen? - Wird die paraproteinämische Keratopathie durch die MM-Therapie beeinflusst? |
| **Medizinprodukte für die Untersuchungen** | Ophthalmologische Diagnostik:   - Optische Kohärenz Tomographie (OCT) - Pentacam - Konfokale Mikrokopie (Rostock Cornea Modul) - Ocular Response Analyzer   Hämatologische Routinediagnostik:   - Whole-body low-dose CT or - Whole-body MRT+ or - Whole-body-PET-CT - Histopathology of the bone marrow - From the blood of the bone marrow:   - Zytologische Befundung   - Durchflusszytometrische Befundung   - Zytogenetische Befundung |
| **Methoden/ Studienablauf** | Erstvorstellung (Gruppen 1 – 3):   - Anamnese (hämatologische, ophthalmologische) - Medikamente - Klinische Untersuchung   Hämatologie (Gruppen 1 - 3):   - Labor (**im Rahmen der Routine Diagnostik**):   - Differentialblutbild   - Elektrolyte: Serumkalzium gesamt, Natrium, Kalium, Phosphat   - Serumkreantinin, Harnsäure, Serumharnstoff, GPT,GOT, ALP, gGT, Billirubin gesamt, CRP, LDH   - IgG, A,M,D,E, Kappa und Lambda-LK im Serum, kappa/Lambda-Ratio, freie Kappa-LK, freie Lambda-LK, freie kappa/lambda-Ratio, Immunfixation im Serum, M-Gradient   - Serumelektrophorese   - Serumalbumin, beta-2-Mikroglobulin   - Quick, INR, apTT, Fibrinogen gesamt   - Hepatitis A-C-Serologie-, HIV-Test, CMV & EBV-PCR   - 24-H-Sammelurin: Kreatinin-Clearance, Albumin im Sammelurin, kappa- und lambda-Leichtketten im Sammelurin, Immunfixation - Low-Dose Ganzkörper CT/Ganzkörper-MRT/Ganzkörper PET-CT (Bei Erstdiagnose oder je nach klinischer Indikation - Bei klinischer Indikation weitere Zielaufnahmen mittels Röntgen/CT/MRT - Knochenmarkspunktion bei Erstdiagnose oder klinischer Indikation   - Zytologie   - Histologie   - Durchflusszytometrie   - Zyotogenetik   Augenklinik:   - Bestimmung der bestkorrigierten Sehschärfe - Spaltlampenuntersuchung, Beschreibung der Hornhauttrübung falls vorhanden - Vorderabschnitts-OCT - Hornhauttopographie und Hornhautdicke (Pentacam) - Konfokale Mikroskopie - Übersichtsfoto des vorderen Augenabschnitt - indirekte Ophthalmoskopie in Miosis - Augeninnendruck-Messung mit Goldmann Applanationstonometrie - Ocular Response Analyzer   Follow-up 3, 6 und 12 Monate nach der Studieneinschluss:  Hämatologie:   - Erfassung der hämatologischen Therapie in Gruppe 3 (MM). - Gruppen 1-3:   - Differentialblutbild   - Elektrolyte: Serumkalzium gesamt, Natrium, Kalium, Phosphat   - Serumkreantinin, Harnsäure, Serumharnstoff, GPT,GOT, ALP,gGT, Billirubin gesamt, CRP, LDH   - IgG,A,M, Kappa und Lambda-LK im Serum, kappa/Lambda-Ratio, freie Kappa-LK, freie Lambda-LK, freie kappa/lambda-Ratio, Immunfixation, M-Gradient   - Serumeletrophorese   - Quick, INR, apTT, Fibrinogen gesamt - 24-H-Sammelurin: Kreatinin-Clearance, Albumin im Sammelurin, kappa- und lambda-Leichtketten im Sammelurin, Immunfixation - Knochenmarkpunktion und Bildgebung nur bei klinischer Indikation   Augenklinik:   - wie bei der Erstvorstellung (Gruppen 1-3) |
| **Studiendauer** | Nach dem positiven Ethikvotum voraussichtlich 36 Monate |
