# Supplementary material for: Prevalence of corneal findings and their interrelation with hematological findings in monoclonal gammopathy
Source: PLoS One. 2022 Oct 31;17(10):e0276048. doi: 10.1371/journal.pone.0276048 (PMC9621422; doi:10.1371/journal.pone.0276048)
Supplement: S2 Protocol — (DOC) [file pone.0276048.s003.doc]

**Inzidenz der paraproteinämischer Keratopathie bei Patienten mit monoklonaler Gammopathie unklarer Signifikanz, smoldering multiplem Myelom und multiplem Myelom.**

**(Incidence of paraproteinemic keratopathie in patients with monoklonal gammopathie of undetermined significance, smoldering multiple myeloma und multiple myeloma.)**

*Finally, we analyzed the prevalence of the paraproteinemic keratopathy instead of the incidence*

**Sponsor:**

Department of Ophthalmology, University Medical Center of the Johannes Gutenberg-University Mainz, Germany

**Studienleiterin:**

Dr. med. Joanna Wasielica-Poslednik

Oberärztin der Klinik

Adresse: Augenklinik und Poliklinik, Langenbeckstr. 1, 55131 Mainz

Telefon +49 6131 176038

E-Mail: joanna.wasielica-poslednik@unimedizin-mainz.de

**Synopsis**

| **Title** | **Incidence of paraproteinemic keratopathy in patients with monoclonal gammopathy of undetermined significance (MGUS), smoldering multiple myeloma (SMM) und multiple myeloma (MM)** |
| --- | --- |
| **Short title** | **Paraproteinemic keratopathy** |
| **Protocol Number** | MZ-MGUS-2016 |
| **Sponsor** | Department of Ophthalmology, University Medical Center of the Johannes Gutenberg-University Mainz, Germany |
| **Study design** | Prospective cohort study |
| **Study population** | Consecutive patients visiting the Department of Hematology within 12 months due to the following diagnoses:  **Group 1**: Patients with MGUS  **Group 2**: Patients with SMM  **Group 3**: Patients with MM |
| **Inclusion criteria** | **Group 1**: **Patients with MGUS**  -serum monoclonal protein < 30 g/l, clonal bone marrow plasma cells <10%, and absence of an end organ damage defined as hypercalcemia, renal insufficiency, anemia, and bone lesions (CRAB features) or amyloidosis that can be attributed to the plasma cell proliferative disorder, paraprotein in urine <500mg/24h  **Group 2**: **Patients with SMM**  Serum monoclonal protein (IgG or IgA) ≥30 g/L or urinary monoclonal protein ≥500 mg/24 h and/or clonal bone marrow plasma cells 10–60%; and: absence of amyloidosis or myeloma deﬁning events (like CRAB features or special biomarkers of malignancy)  **Group 3**: **Patients with MM**  is defined as serum monoclonal protein (IgG or IgA) ≥30 g/l or urinary monoclonal protein ≥500 mg/24 h and/or clonal bone marrow plasma cells ≥10%; and one or more of myeloma deﬁning events (CRAB features or special biomarkers of malignancy, which indicate the high level of activity)  **All groups:**   - Male and female - ≥ 18 years - capable of giving consent to participate in the study |
| **Exclusion criteria** | - condition after bilateral refractive corneal surgery (like LASIK, LASEK, PTK) - corneal and intraocular inflammation; - patients with non-measurable M-protein in urine and serum, asecretory multiple myeloma and plasma cell leukemia - diseases associated with monoclonal gammopathy other than MGUS/SMM/MM |
| **Endpoints** | - How high is the incidence of paraproteinemic keratopathy in patients with MGUS, SMM und MM?   *Finally we analyzed the prevalence of the paraproteinemic keratopathy instead of the incidence*   - Does the morphology of paraproteinemic keratopathy correlate with the type of monoclonal gammopathy ? - Does systemic therapy for MM influence the paraproteinemic keratopathy? |
| **Medical examinations** | **Ophthalmological workup:**   - Best corrected visual acuity (Snellen) - Biomicroscopy on the slit lamp - Fundoscopy - optical coherence tomography (OCT) - Scheimpflug tomography - In vivo confocal laser scanning microscopy (Rostock Cornea Modul) - Photography of the cornea - Goldmann applanation tonometry - Ocular Response Analyzer *(not done)*   **Hematological workup:**   - laboratory tests including differential blood count, electrolytes, serum creatinine, uric acid, serum urea, alanine transaminase (ALT), Aspartate transaminase (AST), alkaline phosphatase (ALP), gamma-glutamyl transferase (GGT), total bilirubin, C-reactive protein (CRP), lactate dehydrogenase (LDH), immunofixation in serum, immunoglobulins (Ig) G, A, M, D, and E, kappa (κ) - and lambda (λ) light chains (LC) in serum, kappa/lambda ratio, free kappa light chains (FLC κ), free lambda light chains (FLC λ), free kappa/lambda light chains ratio, M gradient, serum protein electrophoresis, serum albumin, beta-2-microglobulin, International Normalized Ratio (INR), activated Partial Thromboplastin Time (apTT), total fibrinogen; 24-hour urine collection included: creatinine clearance, albumin, kappa and lambda light chains and immune fixation; bone marrow puncture including histology, cytology, flow cytometry and cytogenetics; low-dose whole-body computed tomography. |
| **Methods** | First visit (groups 1 – 3):   - Anamnesis - medicines - complete hematological and ophthalmological workups (see „Medical examinations“)   3-, 6- and 12-months follow-up:  Hematology:   - Documentation of systemic therapy in group 3 (MM). - Groups 1-3:   - differential blood count   - electrolytes   - serum creatinine, uric acid, serum urea, ALT, AST, ALP, GGT, GPT,GOT, total bilirubin, CRP, LDH   - IgG,A,M, kappa and lambda-light chains in serum, kappa/lambda-Ratio, free kappa-LC, free lambda-LC, free kappa/lambda-ratio, immunfixation, M-gradient   - Serumeletrophoresis   - Quick, INR, apTT, total fibrinogen - 24-hour urine collection: creatinine clearance, albumin and kappa- und lambda-LC in urine collection, immunfixation - Bone marrow puncture   Ophthalmology:   - Like by the first visit |
| **Duration of study** | 36 Months |
