# Supplementary material for: Prevalence of corneal findings and their interrelation with hematological findings in monoclonal gammopathy
Source: PLoS One. 2022 Oct 31;17(10):e0276048. doi: 10.1371/journal.pone.0276048 (PMC9621422; doi:10.1371/journal.pone.0276048)
Supplement: S1 File — (DOCX) [file pone.0276048.s004.docx]

# Ethics application

# (klinische Forschung außerhalb des Geltungsbereichs des AMG)

## Allgemeine Angaben

1. **Datum der Antragstellung:** 26.04.16
2. **Titel des Forschungsvorhabens:**Inzidenz der paraproteinämischen Keratopathie bei Patienten mit monoklonaler Gammopathie unklarer Signifikanz (MGUS), smoldering multiplem Myelom (SMM) und multiplem Myelom (MM).
3. **Verantwortlicher Studienleiter (LKP; Hauptprüfer):**

Dr. med. Joanna Wasielica-Poslednik (Fachärztin für Augenheilkunde, Funktionsoberärztin der Klinik)

1. **Verantwortlicher Sponsor:**
   Augenklinik und Poliklinik, Universitätsmedizin Mainz, Johannes-Gutenberg-Universität Mainz
2. **Handelt es sich um eine multizentrische Studie?**
   Nein
3. **Sonstige Teilnehmer mit Orts- und Berufsangabe**Prof. Dr. med. Walter Lisch, Prof. Dr. med. Matthias Theobald (Direktor der III. Med. Klinik), Dr. med. Alexander Desuki (Assistenzarzt der III. Med. Klinik), PD Dr. med Adrian Gericke (Oberarzt der Klinik), Dr. med. Katharina Bell (Assistenzärztin der Klinik), Veronika Weyer (Statistikerin, IMBEI), Christopher Sittel (Doktorand)
4. **Ort(e) der Durchführung des Forschungsvorhabens:**Augenklinik und Poliklinik, Universitätsmedizin Mainz, Johannes-Gutenberg-Universität Mainz

III. Medizinische Klinik und Poliklinik, Hämatologie, Internistische Onkologie und Pneumologie Johannes-Gutenberg-Universität Mainz

1. **Finanzierung:
   - Kostenträger des Forschungsvorhabens:** Das Forschungsvorhaben wird aus Mitteln der Augenklinik bestritten
   **- Höhe des Probanden-Honorar:** Trifft nicht zu
2. **Wurde die Arzneimittelprüfung der zuständigen Behörde angezeigt?**Trifft nicht zu

**Wurde das Forschungsvorhaben bereits einer anderen Ethik-Komission vorgelegt und wenn ja, mit welchem Ergebnis?**Nein

## Begründung des Forschungsvorhabens

## Objective(s) of the research project

Clarification of the following questions:

(a) What is the incidence of paraproteinemic keratopathy in patients with monoclonal gammopathy of undetermined significance (MGUS) smoldering multiple myeloma (SMM) and multiple myeloma (MM)? Do the frequency and pattern of opacification differ in the above groups?

b) Is paraproteinemic keratopathy influenced by MM therapy?

1. **Reasons for implementation, problem statement:**

Monoclonal gammopathy is a pathological increase of a monoclonal antibody in the serum of patients with or without clinical symptoms. The frequency of occurrence of ocular involvement in the form of corneal opacity, so-called paraproteinemic keratopathy, in patients with hematological diseases such as MGUS, SMM or MM is as yet unknown. An improvement of the ocular symptoms with therapy of the underlying hematological disease has been reported in individual cases. Clinical studies that have investigated the incidence and clinic of ocular and hematological symptoms and their correlation are lacking so far. Within the framework of our study, we would like to clarify the incidence of paraproteinemic keratopathy in patients with MGUS, SMM and MM and whether a prediction of future hematological disease can already be made through the ophthalmological examination.

Translated with www.DeepL.com/Translator (free version)

1. **State of the science:**"Monoclonal gammopathy" refers to a form of proliferation of a monoclonal immunoglobulin that results from degeneration of a B-cell clone. This is primarily a disease of old age.

It is found in about 1-3% of people over 50 years of age.

Criteria for the classification of a monoclonal gammopathy of undetermined significance (MGUS) are a plasma cell infiltration of <10% in the bone marrow, a concentration of paraprotein in the serum of <30g/l and in the urine <500mg/24h and no presence of end organ damage analogous to the IMWG criteria (renal insufficiency, anaemia, bone lesion, hypercalcaemia).

Standing alone, MGUS has no disease value, but it is considered the most frequent precancerous condition for the development of a lymphoproliferative disease (MM). SMM is a stage of disease in which the criteria for multiple myeloma are met, but there is no end-organ damage, analogous to the CRAB criteria. Criteria for the diagnosis of SMM in patients with diagnosed monoclonal gammopathy in urine or serum are a plasma cell concentration in the bone marrow of more than 10% and/or a concentration of paraprotein in serum of >30g/l or in urine of >500mg/24h without the presence of end-organ damage.

Diagnosis of treatment-emergent MM is defined as fulfilled SMM criteria, as well as one or more CRAB criteria: Hypercalcaemia (serum total calcium >2.75mmol/l), renal insufficiency (creatinine clearance <= 40ml/min or serum creatinine >2mg/dl) anaemia (HB <10g/dl absolute or >2.5g/dl difference from the lower normal range) and the presence of osteolytic bone lesions (CRAB+).

Expanded biomarker criteria (SLiM) are as follows:

The concentration of clonal plasma cells in the bone marrow is >60%,

the FLC ratio involved/uninvolved light chain is >100 and more than one focal lesion is evident on whole-body MRI.

The risk of progression is approximately 1-1.5% per year. The initial finding is often incidental to routine serum protein electrophoresis, as many affected patients are initially asymptomatic. Ocular involvement in the form of corneal opacity, so-called paraproteinemic keratopathy, may occur. Due to the high heterogeneity of the clinical picture, paraproteinemic keratopathies are often confused with other corneal diseases, especially dystrophies, and are therefore probably often underestimated. The actual frequency of occurrence of such a change is unknown so far. Such a case of keratopathy was documented by Bürki in 1953 and more recently in the largest study to date of paraproteinaemic keratopathy in MGUS (15 patients) by Lisch, Wasielica-Poslednik and Kivelä. The answer to the question of whether ocular involvement can provide information about the further course of the disease or its progression also remains unknown.

In cases of pronounced corneal damage, corneal transplantation may even be necessary, although it is possible that deposits may reappear after successful transplantation if the haematological disease underlying the clinical picture persists3. Currently, according to haematological guidelines, there is no indication for systemic therapy in MGUS. In the case of marked corneal opacity with impaired visual acuity, treatment of the causative MGUS could be considered.

This study should clarify the incidence of paraproteinemic keratopathy in patients with MGUS or SMM and MM. Furthermore, it can be examined whether differences in frequency and pattern of opacification exist in the individual groups mentioned and whether systemic therapy influences the ocular findings.

Translated with www.DeepL.com/Translator (free version)

1. **Zusammenfassende Darstellung der tierexperimentellen und klinischen, pharmakologisch-toxikologischen Eigenschaften von Prüfsubstanzen/Arzneimitteln, wenn diese studienbedingt eingesetzt werden (Beifügung einer aktuellen Investigator’s Brochure bzw. der Fachinformation bei zugelassenen Arzneimitteln)**
   Trifft nicht zu

## Allgemeine Planung

1. **Summary of the test procedure**

All patients with a diagnosis of MGUS, SMM or MM presenting at the III Medical Clinic will be asked to participate in the study. In case of a positive response, a detailed ophthalmological examination will be performed.

A hematological and ophthalmological anamnesis is taken from all patients during the initial presentation. Special attention is paid to medication. Patients in groups 1-3 undergo routine diagnostics at the III Medical Clinic. Imaging such as whole-body CT/MRI or radiological target images are carried out as part of staging or according to clinical indication. The same applies to bone marrow puncture. In opthalmological diagnostics, the determination of the best-corrected visual acuity, a slit-lamp examination, anterior segment OCT are performed in all three groups. The corneal topography and corneal thickness are determined using Pentacam. Other diagnostic methods used are confocal microscopy, overview photography of the anterior segment, indirect ophthalmoscopy in miosis, intraocular pressure measurement using GAT and the use of an ocular response analyser.

The above examinations are repeated in the MM group after 3, 6 and 12 months.

Translated with www.DeepL.com/Translator (free version)

**Geplanter Beginn und voraussichtliche Dauer des Forschungsvorhabens**

1. Nach positivem Ethikvotum voraussichtlich 36 Monate

1. **Examination of patients**

**Haematology (groups 1-3) (routine examinations at first presentation for the above-mentioned diseases):**

- Differential blood count

-Electrolytes: total serum calcium, sodium, potassium, phosphate.

-Serum creatinine, uric acid, serum urea, GPT, GOT, ALP, gGT, total bilirubin, CRP, LDH

-IgG, A, M, D, E, kappa and lambda LCs in serum, kappa/lambda ratio, free kappa LCs, free lambda LCs, free kappa/lambda ratio, immune fixation in serum, M-gradient

-Serum electrophoresis

-Serum albumin, beta-2-microglobulin

-Quick, INR, apTT, total fibrinogen

-Hepatitis A-C serology, HIV test, CMV & EBV PCR

-24-hour collection urine: creatinine clearance, albumin in collection urine, kappa and lambda light chains in collection urine, immunofixation

- Low-dose whole-body CT/whole-body MRI/whole-body PET-CT (depending on indication)

- If clinically indicated, further target images by X-ray/CT/MRI

- Bone marrow puncture at initial diagnosis or depending on clinical indication

Ophthalmology (groups 1-3):
- Determination of the best corrected visual acuity

- Slit lamp examination, description of corneal opacity if present

- Anterior segment OCT

- Corneal topography and thickness (Pentacam)

- Confocal microscopy

- Overview photo of the anterior segment

- Indirect ophthalmoscopy in miosis

- Intraocular pressure measurement with GAT

- Ocular Response Analyzer

1. **It is a:**

prospective cohort study

1. **Darstellung der Studienart**

Prospektive Kohortenstudie

1. **Bei Forschungsvorhaben, bei denen Arzneimittel eingesetzt werden, angeben, ob die Arzneimittel**

Es handelt sich nicht um eine Arzneimittelprüfung.
Für die GAT werden routinemäßig die Augen mit einer festen Kombination von Oxybuprocain 0,4%/Fluoreszein 0,08% getropft. Diese Mischung wird in der Apotheke der Universitätmedizin vorbereitet und in der Augenklinik Routine zur IOD-Messungen verwendet

1. **Angaben zum Monitoring und Audit** Nicht vorgesehen
2. **Bei Prüfungen mit Medizinprodukten angeben, ob eine CE-Kennzeichnung vorliegt [beifügen]]**

Trifft nicht zu.

## Selection of patients

1. **General information:**

>18 years of age, basically independent of gender, patient is able to participate in the study, patient has consented to study participation.

1. **Inclusion criteria for patients**

**Group1**

Patients with MGUS

-Patients with monoclonal gammopathy in urine and/or serum

-No evidence of end-organ damage analogous to IMWG criteria (renal failure, anaemia, bone lesion, hypercalcaemia).

-plasma cells <10% in bone marrow and paraprotein in serum <30g/l and paraprotein in urine <500mg/24h

**Group 2**

Patients with SMM

-Patients with monoclonal gammopathy in urine and/or serum

-more than 10% plasma cells in bone marrow and/or paraprotein of >30g/l in serum and/or paraprotein in urine >500mg/24h

-no evidence of end-organ damage analogous to IMWG criteria

-no positive biomarker criteria

**Group 3**

Patients with treatment-naive MM

-before initiation of systemic therapy

-SMM+

-either CRAB+ (see II 3) and/or biomarker criteria+ (see also II 3)

1. **Exclusion criteria for patients**

- Condition after refractive corneal surgery (e.g. LASIK, LASEK, PTK)

-Patients with a monoclonal gammopathy due to an underlying haematological disease, not corresponding to an SMM or MM.

- Exclusion Patients with asecretory multiple myeloma

1. **Werden Patienten eingeschlossen, bei denen Zweifel an der Einsichtsfähigkeit bestehen, bzw. die Einsichtsfähigkeit nicht vorliegt?** Nein
2. **Description of the inclusion in the research project**

Consecutive patients who present at the II. Med. Clinic within 12 months with the diagnoses mentioned in point 2 will be asked about participation in the study.

1. **Angaben, ob und warum Patienten/Probanden nach Aufnahme in das Forschungsvorhaben ersetzt werden**Nicht vorgesehen.

## Ablauf

1. **Angabe über die Einnahme von Prüfsubstanzen/ Arzneimittel/
   medikamentöser Begleittherapie** Trifft nicht zu
2. **Indication of study-related diagnostic and therapeutic measures:**

Ophthalmic diagnostics:

Optical Coherence Tomography (OCT), Pentacam, Confocal Microscopy (HRT II + Rostock Cornea Module), Ocular Response Analyzer.

Haematological diagnostics:

Whole-body low-dose CT and/or whole-body MRI+ and/or whole-body PET-CT for initial diagnosis as part of the standard, target radiography/MRI/CT for clinical indication, histopathological examination of a bone marrow specimen, from bone marrow blood: -cytologicay, -flow cytometry, -cytogenetics

1. **Beschreibung der Behandlung der Kontrollgruppe**

Ophthalmologische Diagnostik wie im Punkt 2.

1. **Angaben darüber, ob eine Vor- oder Begleitmedikation modifiziert oder abgesetzt werden soll:** Trifft nicht zu
2. **Angaben zu speziellen Belastungstests (pharmakologische, körperliche, mentale Belastung) mit Darstellung der Abbruchkriterien**

Bis auf Applanationstonometrie ausschließlich non-kontakt Untersuchungen, geringer Zeitaufwand, geringe Belastung.

1. **Kontrolle des Gesundheitszustands der Patienten vor, während und nach dem Forschungsvorhaben**

Erstvorstellung, dann Follow-up nach 3,6 und 12 Monaten nach der Erstvorstellung in der MM-Gruppe.

1. **Regeln zum Abbruch des Forschungsvorhabens für den Einzelnen, wie auch für die Gesamtstudie**

Bei jeglicher Gesundheitsgefährdung des Einzelnen, sofortiger Abbruch.

1. **Regeln für die Fortführung einer Therapie nach Studienende**Trifft nicht zu.

## Feststellung der Wirksamkeit Trifft nicht zu.

## Statistik und Auswertung

1. **Auswerten der Prüfgrößen mit Darstellung der statistischen Methoden**Bezüglich der statistischen Methoden, verweisen wir auf das angehängte Dokument: „Statistische Methoden und Fallzahlkalkulation“.
2. **Angaben über die Erstellung der Prüfbögen**Eingabe/ Auswertung der anonymisierten Daten in Excel-Tabellen.
3. **Angaben über Zwischenauswertungen** Nein
4. **Angaben über den Umgang mit Patienten-/Probandendaten**

Anonymisiert und vertraulich.

## Ethisch-rechtliche Aspekte

1. **Angaben darüber, dass die bestehenden Gesetze, Vorschriften und Richtlinien (siehe Nachfolgende Auflistung) beachtet und eingehalten werden: z.B. die einschlägigen Paragraphen des AMG und MPG (sowie entsprechende Ausführungsverordnungen), die Fassung der Deklaration des Weltärztebundes über biomedizinische Forschung am Menschen von 1996, die Berufsordnung der deutschen Ärzte, die Strahlenschutzverordnung und die Röntgenverordnung, das Medizinproduktgesetz und die entsprechenden Verordnungen, Datenschutzgesetze:**

Werden eingehalten.

1. **Angaben zu folgenden Fragen:**
2. **Dient das Forschungsvorhaben**

- **unmittelbar dem Interesse des Patienten?** Ja
- **einem rein wissenschaftlichen Ziel?** Nein
- **der künftigen Entwicklung von diagnostischen und therapeuthischen Verfahren?** Ja
- **der Gewinnung von Erkenntnissen über Pathogenese und Prognose von Krankheiten?** Ja
- **der Gewinnung von Erkenntnissen über sozialmedizinische Probleme?** Nein

1. **Bestehen Risiken für die Probanden/Patienten? Wenn ja, welche?**Ein sehr geringes Risiko der vorübergehenden Hornhautschädigung bzw. einer allergischen Reaktion/Unverträglichkeit gegen anästhetische Augentropfen (notwendig für die Goldmann/Perkins Applanationstonometrie)
2. **Entstehen für die Probanden/Patienten zusätzliche Belastungen? Wenn ja, welche?** Nein
3. **Welche typischen und seltenen unerwünschten Wirkungen der Prüfsubstanz/Therapiemaßnahmen sind zu erwarten?**Trifft nicht zu
4. **Welche Interaktionen der Prüfsubstanz mit eingenommenen Medikamenten können entstehen?** Trifft nicht zu
5. **Welche Komplikationen können während des Forschungsvorhabens auftreten?** Siehe Punkt 2b
6. **Erfassung und Behandlung von Komplikationen und unerwünschten Wirkungen:**
7. **Definition der Schweregrade der unerwünschten Wirkungen**

Es kann in sehr seltenen Fällen eine oberflächliche Verletzung der Hornhaut durch die Applanationstonometrie hervorgerufen werden (sog. Erosio corneae). Diese ist schmerzhaft und heilt i.d.R. innerhalb von 2 Tagen ab. Wenn unbehandelt kann dies in seltenen Fällen zur Hornhaut-Entzündung führen.

s. 2B

1. **Wie werden die Komplikationen und unerwünschten Wirkungen erkannt?** Durch die Spaltlampenuntersuchung
2. **Wann und an wen erfolgt Mitteilung?** An die Studienärzte, Hausarzt/Augenarzt
3. **Wie werden die Komplikationen und unerwünschten Wirkungen behandelt, wie lange werden sie kontrolliert?**Mit antibiotischer Augensalbe und Pflege, in der Augenklinik in der klinischen Routine.
4. **Wie können die Belastungen, Komplikationen und unerwünschen Wirkungen minimiert werden?**Bei erfahrenen Studienärzten minimales Risiko
5. **Angabe von Vorsichtsmaßnahmen**Spaltlampenuntersuchung
6. **Welche Vorteile entstehen für die Probanden/Patienten durch die Teilnahme an dem Prüfvorhaben?**

Ausführliche ophthalmologische Untersuchung. Feststellung, ob eine paraproteinämische Keratopathie bzw. Handlungsbedarf besteht.

1. **Besteht entsprechender Versicherungsschutz?**

Nein

1. **Ist der Prüfungsleiter entsprechend GCP-ICH Richtlinien informiert?**

Ja

1. **Wurden ähnliche Forschungsvorhaben oder vergleichbare Untersuchungen mit der Prüfsubstanz bereits durchgeführt? Wenn ja, mit welchem Ergebnis?** Nicht zutreffend
2. **Angaben darüber, dass in mündlicher und schriftlicher Form aufgeklärt wird. Eine schriftli­che Probanden/Patienteninformation ist beizufügen, in der in allgemein ver­ständlicher Form über Wesen, Ablauf und Ziel des Forschungsvorhabens, studienbe­dingte Änderungen einer Vormedikation, studienbedingte diagnostische Maßnahmen und Belastungstests, Placebo-Gabe während einer Auswasch- und/oder Behand­lungsphase, Therapie in der Vergleichsgruppe und Standardtherapie, Randomisierung, erwarteten Nutzen (z.B. persönlicher Nutzen vorhanden, wenn ja welcher), Komplika­ti­onen, unerwünschte Wirkungen, Dauer der Studie, Fortführung der Therapie am Stu­dienende, Blutentnahmen mit Angabe der Menge, Versicherungsanschrift, Policen­nummer, Telefonnummer, Höchstsumme und die wichtigsten Versicherungsobliegen­heiten informiert wird.**Siehe Probanden-/Patienteninformation
3. **Einwilligungserklärung**

Siehe Probanden-/Patienteninformation

1. **In der schriftlichen Einwilligungserklärung muss auf die Proban­den/Patienteninforma­tion verwiesen werden. Es müssen die im Vorwort aufgeführten Inhalte bestätigt wer­den.**

Siehe Anlage

1. **Angaben darüber, ob Patienten, bei denen Zweifel an der Einsichtsfähigkeit bestehen, in das Forschungsvorhaben aufgenommen werden. In diesem Fall muss ein unabhän­giger Sachverständiger (Facharzt/-ärztin für Psychiatrie), der nicht mit dem For­schungsvorhaben befasst ist, zuvor das Vorhandensein der Einsichtsfähigkeit bestätigt haben.**

Werden nicht eingeschlossen

1. **Angaben darüber, ob Patienten mit eingeschränkter oder nicht vorhandener Einwilli­gungsfähigkeit (z.B. akute schwerwiegende Erkrankung; Bewusstseinseinschränkung; Bewusstlosigkeit; Demenzerkrankungen; Patienten, die unter gesetzlicher Betreuung stehen) in das Forschungsvorhaben aufgenommen werden sollen. In diesem Fall muss eine ausführliche Begründung erfolgen, in der dargelegt wird, dass im Sinne ei­ner sog. Ausnahmeregelung die Bedingungen eingehalten werden, die durch die Stel­lung­nahme der Zentralen Ethik-Kommission bei der BÄK sowie in der europäischen Bio­ethik-Konvention festgelegt sind. Die vorgesehene Forschung muss dem unmittel­baren Nutzen für den Patienten (individueller Nutzen) dienen. Liegt bei gesetzlich be­treuten Patienten eine Grundeinsichtsfähigkeit vor, muss neben der Zustimmung des Betreuers auch der Patient seine Einwilligung erklären**.

Werden nicht eingeschlossen

1. **Angaben darüber,**
2. **ob ein Proband-/Patientenausweis ausgestellt wird**  Nein
3. **ob der weiterbehandelnde Arzt (in der Regel der Hausarzt) über die Teilnahme seines Patienten an dem Forschungsvorhaben informiert wird** Ja, auf Wunsch des Patienten.
4. **dass der Prüfarzt über die notwendige fachliche und persönliche Qualifikation, Erfahrung, Personal, Zeit und Einrichtung verfügt, um das Forschungsvorhaben in der beantragten Weise durchzuführen (curriculum vitae)**

Studienleiterin und alle beteiligten Ärzte sind Studien-/Prüfärzte (Kopie anbei) mit Erfahrung in der Durchführung von klinischen Studien.

1. **dass der Leiter der Einrichtung, an der das Forschungsvorhaben realisiert werden soll, mit der Druchführung einverstanden ist**

Siehe Einverständniserklärung des Klinikdirektors.
