# Supplementary material for: Prevalence of corneal findings and their interrelation with hematological findings in monoclonal gammopathy
Source: PLoS One. 2022 Oct 31;17(10):e0276048. doi: 10.1371/journal.pone.0276048 (PMC9621422; doi:10.1371/journal.pone.0276048)
Supplement: S2 File — (DOCX) [file pone.0276048.s005.docx]

# Ethik- Antragstellung

# (klinische Forschung außerhalb des Geltungsbereichs des AMG)

## Allgemeine Angaben

1. **Datum der Antragstellung:** 26.04.16
2. **Titel des Forschungsvorhabens:**Inzidenz der paraproteinämischen Keratopathie bei Patienten mit monoklonaler Gammopathie unklarer Signifikanz (MGUS), smoldering multiplem Myelom (SMM) und multiplem Myelom (MM).
3. **Verantwortlicher Studienleiter (LKP; Hauptprüfer):**

Dr. med. Joanna Wasielica-Poslednik (Fachärztin für Augenheilkunde, Funktionsoberärztin der Klinik)

1. **Verantwortlicher Sponsor:**
   Augenklinik und Poliklinik, Universitätsmedizin Mainz, Johannes-Gutenberg-Universität Mainz
2. **Handelt es sich um eine multizentrische Studie?**
   Nein
3. **Sonstige Teilnehmer mit Orts- und Berufsangabe**Prof. Dr. med. Walter Lisch, Prof. Dr. med. Matthias Theobald (Direktor der III. Med. Klinik), Dr. med. Alexander Desuki (Assistenzarzt der III. Med. Klinik), PD Dr. med Adrian Gericke (Oberarzt der Klinik), Dr. med. Katharina Bell (Assistenzärztin der Klinik), Veronika Weyer (Statistikerin, IMBEI), Christopher Sittel (Doktorand)
4. **Ort(e) der Durchführung des Forschungsvorhabens:**Augenklinik und Poliklinik, Universitätsmedizin Mainz, Johannes-Gutenberg-Universität Mainz

III. Medizinische Klinik und Poliklinik, Hämatologie, Internistische Onkologie und Pneumologie Johannes-Gutenberg-Universität Mainz

1. **Finanzierung:
   - Kostenträger des Forschungsvorhabens:** Das Forschungsvorhaben wird aus Mitteln der Augenklinik bestritten
   **- Höhe des Probanden-Honorar:** Trifft nicht zu
2. **Wurde die Arzneimittelprüfung der zuständigen Behörde angezeigt?**Trifft nicht zu

**Wurde das Forschungsvorhaben bereits einer anderen Ethik-Komission vorgelegt und wenn ja, mit welchem Ergebnis?**Nein

## Begründung des Forschungsvorhabens

## Ziel(e) des Forschungsvorhabens

## Klärung folgender Fragestellungen:

1. Wie ist die Inzidenz der paraproteinämischen Keratopathie bei Patienten mit monoklonaler Gammopathie unklarer Signifikanz (MGUS) smoldering multiplem Myelom (SMM) und multiplem Myelom (MM)? Unterscheiden sich Häufigkeit und Trübungsmuster in den o.g. Gruppen?
2. Wird die paraproteinämische Keratopathie durch die MM-Therapie beeinflusst?
3. **Gründe für die Durchführung, Problemdarstellung:**

Die monoklonale Gammopathie bezeichnet eine pathologische Vermehrung eines monoklonalen Antikörpers im Serum von Patienten ohne klinische Symptomatik. Die Häufigkeit des Auftretens einer Augenbeteiligung in Form einer Hornhauttrübung, sog. paraproteinämische Keratopathie, bei Patienten mit hämatologischen Erkrankungen wie MGUS, SMM oder MM ist bisher unbekannt. Eine Verbesserung der okulären Symptome bei einer Therapie der grundliegenden hämatologischen Erkrankung wurde in Einzelfällen berichtet. Es fehlen bisher klinische Studien, die die Inzidenz und Klinik der okulären und hämatologischen Symptome und deren Zusammenhang untersucht haben. Im Rahmen unserer Studie möchten wir klären, wie hoch die Inzidenz einer paraproteinämischen Keratopathie bei Patienten mit MGUS, SMM und MM ist und ob sich durch die ophthalmologische Untersuchung bereits eine Vorhersage auf eine zukünftige hämatologische Erkrankung treffen lässt.

1. **Stand der Wissenschaft:**„Monoklonale Gammopathie“ bezeichnet eine Form der Vermehrung eines monoklonalen Immunglobulins, welche durch Entartung eines B-Zell-Klones entsteht. Hierbei handelt es sich in erster Linie um eine Erkrankung des Alters.
   Sie findet sich bei etwa 1-3% von Personen über 50 Jahren^[[1]](#footnote-1)^.
   Kriterien für die Klassifikation einer monoklonalen Gammopathie unklarer Signifikanz (MGUS) sind eine Plasmazellinfiltration von <10% im Knochenmark, eine Konzentration des Paraproteins im Serum von <30g/l und im Urin <500mg/24h und kein Vorliegen eines Endorganschadens analog der IMWG-Kriterien (Niereninsuffizienz, Anämie, Knochenläsion, Hyperkalzämie).
   Alleinstehend hat eine MGUS keinen Krankheitswert, allerdings gilt sie als häufigste Präkanzerose für die Entstehung einer lymphoproliferativen Erkrankung (Multiples Myelom/MM). Das smoldering multiples Myelom (SMM) stellt ein Erkrankungsstadium dar, bei dem die Kriterien zum Vorliegen eines multiplen Myeloms zwar erfüllt werden, jedoch kein Endorganschaden vorliegt, analog den CRAB-Kriterien.^[[2]](#footnote-2)^ Kriterien für die Diagnostik eines SMM bei Patienten mit diagnostizierter monoklonaler Gammopathie im Urin oder Serum sind eine Plasmazellkonzentration im Knochenmark von mehr als 10% und/oder eine Konzentration des Paraproteins im Serum von >30g/l oder im Urin von >500mg/24h ohne Vorliegen eines Endorganschadens.
   Als Diagnostik eines Behandlungspflichtigen MM gelten erfüllte SMM-Kriterien, sowie ein oder mehrere CRAB-Kriterien: Hyperkalzämie (Serumkalzium gesamt >2,75mmol/l), Niereninsuffizienz (Kreatinin-Clearance <= 40ml/min oder Serumkreatinin >2mg/dl) Anämie (HB <10g/dl absolut oder >2,5g/dl Differenz zum unteren Normbereich) und das Vorhandensein von osteolytischen Knochenläsionen (CRAB+).
   Erweitert gelten folgende Biomarker-Kriterien (SLiM):
   Die Konzentration der klonalen Plasmazellen im Knochenmark beträgt >60%,
   die FLC-Ratio involved/uninvolved Leichtkette ist >100 und es ist mehr als eine fokale Läsion im Ganzkörper MRT ersichtlich.
   Die Gefahr einer Progression liegt bei ca. 1-1,5% pro Jahr. ^[[3]](#footnote-3)^ Der Erstbefund entsteht häufig zufällig bei einer routinemäßigen Serumproteinelektrophorese, da viele betroffene Patienten zunächst keine Symptome aufweisen.^[[4]](#footnote-4)^ Okuläre Beteiligung in Form einer Hornhauttrübung, sog. paraproteinämische Keratopathie, kann auftreten. Aufgrund hoher Heterogenität des klinischen Bildes werden die paraproteinämischen Keratopathien oft mit anderen Hornhauterkrankungen, v.a. Dystrophien verwechselt und vermutlich dadurch oft unterschätzt. Die tatsächliche Häufigkeit des Auftretens einer solchen Veränderung ist bisher unbekannt.^[[5]](#footnote-5)^ Dokumentiert wurde ein solcher Fall der Keratopathie von Bürki im Jahre 1953^[[6]](#footnote-6)^ und in neuerer Zeit im Rahmen der bisher größten Untersuchung von paraproteinämischer Keratopathie bei MGUS (15 Patienten) durch Lisch, Wasielica-Poslednik und Kivelä.^[[7]](#footnote-7)^ Unbekannt bleibt auch die Antwort auf die Frage, ob die okuläre Beteiligung über den weiteren Verlauf der Erkrankung oder ihrer Progression eine Aussage geben kann.

Bei ausgeprägten Hornhautschäden kann sogar eine Hornhauttransplantation notwendig werden, allerdings ist es hierbei möglich, dass es nach erfolgreicher Transplantation erneut zu Ablagerungen kommen kann, sofern die dem Krankheitsbild zugrundeliegende hämatologische Erkrankung weiterhin besteht^3^. Zur Zeit besteht nach hämatologischen Leitlinien keine Indikation zur systemischen Therapie bei MGUS. Im Fall einer ausgeprägten Hornhauttrübung mit Beeinträchtigung der Sehschärfe könnte die Behandlung der ursächlichen MGUS erwogen werden.

Im Rahmen dieser Studie sollte die Inzidenz der paraproteinämischen Keratopathie bei Patienten mit MGUS oder SMM und MM geklärt werden. Weiterhin kann in diesem Rahmen überprüft werden, ob Unterschiede in Häufigkeit und Trübungsmuster in den einzelnen genannten Gruppen existieren und ob die systemische Therapie den Augenbefund beeinflusst.

1. **Zusammenfassende Darstellung der tierexperimentellen und klinischen, pharmakologisch-toxikologischen Eigenschaften von Prüfsubstanzen/Arzneimitteln, wenn diese studienbedingt eingesetzt werden (Beifügung einer aktuellen Investigator’s Brochure bzw. der Fachinformation bei zugelassenen Arzneimitteln)**
   Trifft nicht zu

## Allgemeine Planung

1. **Zusammenfassende Darstellung des Prüfablaufs**

Alle Patienten mit der Diagnose einer MGUS, SMM oder MM, die in der III. Medizinischen Klinik vorgestellt werden, werden zur Teilnahme an der Studie angesprochen. Bei positiver Antwort wird eine ausführliche augenärztliche Untersuchung durchgeführt.

Bei allen Patienten wird im Rahmen der Erstvorstellung eine hämatologische und ophthalmologische Anamnese erhoben. Besonderes Augenmerk gilt hierbei der Medikation. Mit den Patienten der Gruppen 1-3 wird in der III. Medizinischen Klinik eine Routinediagnostik durchgeführt. Bildgebungen wie Ganzkörper-CT/MRT oder radiologische Zielaufnahmen werden im Rahmen des Stagings bzw. nach klinischer Indikation durchgeführt. Gleiches gilt für eine Knochenmarkspunktion. In der opthalmologischen Diagnostik erfolgt in allen drei Gruppen die Bestimmung der bestkorrigierten Sehschärfe, eine Spaltlampenuntersuchung, Vorderabschnitts-OCT. Mittels Pentacam wird die Hornhauttopographie und Hornhautdicke bestimmt. Weitere Möglichkeiten der Diagnostik, welche zur Anwendung kommen, sind die konfokale Mikroskopie, Übersichtsfotographie des vorderen Augenabschnitts, eine indirekte Opthalmoskopie in Miosis, sowie Augeninnendruck Messung mittels GAT und dem Einsatz eines Ocular Response Analyzers.

Die o.g. Untersuchungen werden in der MM-Gruppe nach 3, 6, und 12 Monaten wiederholt.

1. **Geplanter Beginn und voraussichtliche Dauer des Forschungsvorhabens**
2. Nach positivem Ethikvotum voraussichtlich 36 Monate

1. **Untersuchung an Patienten**Hämatologie: (Gruppen 1-3) (Routineuntersuchungen bei der Erstvorstellung bei o.g. Krankheiten)
   - Differentialblutbild
   -Elektrolyte: Serumkalzium gesamt, Natrium, Kalium, Phosphat
   -Serumkreatinin, Harnsäure, Serumharnstoff, GPT, GOT, ALP, gGT, Bilirubin gesamt, CRP, LDH
   -IgG, A, M, D, E, Kappa- und Lambda-LK im Serum, Kappa/Lambda-Ratio, freie Kappa-LK, freie Lambda-LK, freie Kappa/Lambda-Ratio, Immunfixation im Serum, M-Gradient
   -Serumelektrophorese
   -Serumalbumin, beta-2-Mikroglobulin
   -Quick, INR, apTT, Fibrinogen gesamt
   -Hepatitis A-C-Serologie, HIV-Test, CMV & EBV-PCR
   -24-h-Sammelurin: Creatinin-Clearance, Albumin im Sammelurin, Kappa- und Lambda- Leichtketten im Sammelurin, Immunfixation
   - Low-Dose Ganzkörper CT/Ganzkörper MRT/ Ganzkörper PET-CT (je nach Indikation
   - Bei klinischer Indikation weitere Zielaufnahmen mittels Röntgen/CT/MRT
   - Knochenmarkspunktion bei Erstdiagnose bzw. je nach klinischer Indikation

   Augenklinik: (Gruppen 1-3)
   - Bestimmung der bestkorrigierten Sehschärfe
   - Spaltlampenuntersuchung, Beschreibung der Hornhauttrübung falls vorhanden
   - Vorderabschnitts-OCT
   - Hornhauttopographie und Hornhautdicke (Pentacam)
   - Konfokale Mikroskopie
   - Übersichtsfoto des vorderen Augenabschnitts
   - indirekte Ophthalmoskopie in Miosis
   - Augeninnendruck-Messung mit GAT
   - Ocular Response Analyzer
2. **Es handelt sich um eine:**

Prospektive Kohortenstudie

1. **Darstellung der Studienart**

Prospektive Kohortenstudie

1. **Bei Forschungsvorhaben, bei denen Arzneimittel eingesetzt werden, angeben, ob die Arzneimittel**

Es handelt sich nicht um eine Arzneimittelprüfung.
Für die GAT werden routinemäßig die Augen mit einer festen Kombination von Oxybuprocain 0,4%/Fluoreszein 0,08% getropft. Diese Mischung wird in der Apotheke der Universitätmedizin vorbereitet und in der Augenklinik Routine zur IOD-Messungen verwendet

1. **Angaben zum Monitoring und Audit** Nicht vorgesehen
2. **Bei Prüfungen mit Medizinprodukten angeben, ob eine CE-Kennzeichnung vorliegt [beifügen]]**

Trifft nicht zu.

## Auswahl der Patienten

1. **Allgemeine Angaben**>18 Jahre alt, grundsätzlich unabhängig vom Geschlecht, Patient ist in der Lage am Studienablauf teilzunehmen, Patient hat zur Studienteilnahme eingewilligt.
2. **Einschlusskriterien für Patienten

   Gruppe1
   Patienten mit MGUS**
    -Patienten mit monoklonaler Gammopathie im Urin und/oder Serum
   -kein Hinweis auf einen Endorganschaden analog der IMWG-Kriterien (Niereninsuffizienz, Anämie, Knochenläsion, Hyperkalzämie)
   -Plasmazellen <10% im Knochenmark und Paraprotein im Serum <30g/l und Paraprotein im Urin <500mg/24h

   **Gruppe 2
   Patienten mit SMM**
   -Patienten mit monoklonaler Gammopathie im Urin und/oder Serum
   -mehr als 10% Plasmazellen im Knochenmark und/oder Paraprotein von >30g/l im Serum und/oder Paraprotein im Urin >500mg/24h
   -kein Hinweis auf einen Endorganschaden analog der IMWG-Kriterien
   -keine positiven Biomarker-Kriterien


   **Gruppe 3
   Patienten mit behandlungspflichtigem MM**
   -vor Beginn einer Systemtherapie
   -SMM+
   -Entweder CRAB+ (siehe II 3) und/oder Biomarker-Kriterien+ (siehe ebenfalls II 3)
3. **Ausschlusskriterien für Patienten**- Zustand nach refraktivem Hornhaut-Eingriff (z.B. LASIK, LASEK, PTK)

-Patienten mit einer monoklonalen Gammopathie aufgrund einer hämatologischen Grunderkrankung, nicht einem SMM oder MM entsprechend
- Ausschluss Patienten mit asekretorischem multiplen Myelom

1. **Werden Patienten eingeschlossen, bei denen Zweifel an der Einsichtsfähigkeit bestehen, bzw. die Einsichtsfähigkeit nicht vorliegt?** Nein
2. **Darstellung der Aufnahme in das Forschungsvorhaben**Konsekutive Patienten, die innerhalb von 12 Monaten mit den in Punkt 2 genannten Diagnosen in der II. Med. Klinik vorstellig werden, werden auf die Teilnahme an der Studie angesprochen.
3. **Angaben, ob und warum Patienten/Probanden nach Aufnahme in das Forschungsvorhaben ersetzt werden**Nicht vorgesehen.

## Ablauf

1. **Angabe über die Einnahme von Prüfsubstanzen/ Arzneimittel/
   medikamentöser Begleittherapie** Trifft nicht zu
2. **Angabe über studienbedingte diagnostische und therapeuthische Maßnahmen:**

Ophthalmologische Diagnostik:
Optische Kohärenz Tomographie (OCT), Pentacam, Konfokale Mikroskopie (HRT II + Rostock Cornea Modul), Ocular Response Analyzer

Hämatologische Diagnostik:
Ganzkörper Low-Dose CT und/oder Ganzkörper MRT+ und/oder Ganzkörper-PET-CT bei Erstdiagnose im Rahmen des Standard, Zielaufnahmen mittels Röntgen/MRT/CT bei klinischer Indikation, Histopathologische Untersuchung eines Knochenmarktrepanats, aus Knochenmarksblut: -zytologische Befundung, – Durchflusszytometrische Befundung, -Zytogenetische Befundung

1. **Beschreibung der Behandlung der Kontrollgruppe**
2. Ophthalmologische Diagnostik wie im Punkt 2.
3. **Angaben darüber, ob eine Vor- oder Begleitmedikation modifiziert oder abgesetzt werden soll:** Trifft nicht zu
4. **Angaben zu speziellen Belastungstests (pharmakologische, körperliche, mentale Belastung) mit Darstellung der Abbruchkriterien**

Bis auf Applanationstonometrie ausschließlich non-kontakt Untersuchungen, geringer Zeitaufwand, geringe Belastung.

1. **Kontrolle des Gesundheitszustands der Patienten vor, während und nach dem Forschungsvorhaben**

Erstvorstellung, dann Follow-up nach 3,6 und 12 Monaten nach der Erstvorstellung in der MM-Gruppe.

1. **Regeln zum Abbruch des Forschungsvorhabens für den Einzelnen, wie auch für die Gesamtstudie**

Bei jeglicher Gesundheitsgefährdung des Einzelnen, sofortiger Abbruch.

1. **Regeln für die Fortführung einer Therapie nach Studienende**Trifft nicht zu.

## Feststellung der Wirksamkeit Trifft nicht zu.

## Statistik und Auswertung

1. **Auswerten der Prüfgrößen mit Darstellung der statistischen Methoden**Bezüglich der statistischen Methoden, verweisen wir auf das angehängte Dokument: „Statistische Methoden und Fallzahlkalkulation“.
2. **Angaben über die Erstellung der Prüfbögen**Eingabe/ Auswertung der anonymisierten Daten in Excel-Tabellen.
3. **Angaben über Zwischenauswertungen** Nein
4. **Angaben über den Umgang mit Patienten-/Probandendaten**

Anonymisiert und vertraulich.

## Ethisch-rechtliche Aspekte

1. **Angaben darüber, dass die bestehenden Gesetze, Vorschriften und Richtlinien (siehe Nachfolgende Auflistung) beachtet und eingehalten werden: z.B. die einschlägigen Paragraphen des AMG und MPG (sowie entsprechende Ausführungsverordnungen), die Fassung der Deklaration des Weltärztebundes über biomedizinische Forschung am Menschen von 1996, die Berufsordnung der deutschen Ärzte, die Strahlenschutzverordnung und die Röntgenverordnung, das Medizinproduktgesetz und die entsprechenden Verordnungen, Datenschutzgesetze:**

Werden eingehalten.

1. **Angaben zu folgenden Fragen:**
2. **Dient das Forschungsvorhaben**

- **unmittelbar dem Interesse des Patienten?** Ja
- **einem rein wissenschaftlichen Ziel?** Nein
- **der künftigen Entwicklung von diagnostischen und therapeuthischen Verfahren?** Ja
- **der Gewinnung von Erkenntnissen über Pathogenese und Prognose von Krankheiten?** Ja
- **der Gewinnung von Erkenntnissen über sozialmedizinische Probleme?** Nein

1. **Bestehen Risiken für die Probanden/Patienten? Wenn ja, welche?**Ein sehr geringes Risiko der vorübergehenden Hornhautschädigung bzw. einer allergischen Reaktion/Unverträglichkeit gegen anästhetische Augentropfen (notwendig für die Goldmann/Perkins Applanationstonometrie)
2. **Entstehen für die Probanden/Patienten zusätzliche Belastungen? Wenn ja, welche?** Nein
3. **Welche typischen und seltenen unerwünschten Wirkungen der Prüfsubstanz/Therapiemaßnahmen sind zu erwarten?**Trifft nicht zu
4. **Welche Interaktionen der Prüfsubstanz mit eingenommenen Medikamenten können entstehen?** Trifft nicht zu
5. **Welche Komplikationen können während des Forschungsvorhabens auftreten?** Siehe Punkt 2b
6. **Erfassung und Behandlung von Komplikationen und unerwünschten Wirkungen:**
7. **Definition der Schweregrade der unerwünschten Wirkungen**

Es kann in sehr seltenen Fällen eine oberflächliche Verletzung der Hornhaut durch die Applanationstonometrie hervorgerufen werden (sog. Erosio corneae). Diese ist schmerzhaft und heilt i.d.R. innerhalb von 2 Tagen ab. Wenn unbehandelt kann dies in seltenen Fällen zur Hornhaut-Entzündung führen.

s. 2B

1. **Wie werden die Komplikationen und unerwünschten Wirkungen erkannt?** Durch die Spaltlampenuntersuchung
2. **Wann und an wen erfolgt Mitteilung?** An die Studienärzte, Hausarzt/Augenarzt
3. **Wie werden die Komplikationen und unerwünschten Wirkungen behandelt, wie lange werden sie kontrolliert?**Mit antibiotischer Augensalbe und Pflege, in der Augenklinik in der klinischen Routine.
4. **Wie können die Belastungen, Komplikationen und unerwünschen Wirkungen minimiert werden?**Bei erfahrenen Studienärzten minimales Risiko
5. **Angabe von Vorsichtsmaßnahmen**Spaltlampenuntersuchung
6. **Welche Vorteile entstehen für die Probanden/Patienten durch die Teilnahme an dem Prüfvorhaben?**

Ausführliche ophthalmologische Untersuchung. Feststellung, ob eine paraproteinämische Keratopathie bzw. Handlungsbedarf besteht.

1. **Besteht entsprechender Versicherungsschutz?**

Nein

1. **Ist der Prüfungsleiter entsprechend GCP-ICH Richtlinien informiert?**

Ja

1. **Wurden ähnliche Forschungsvorhaben oder vergleichbare Untersuchungen mit der Prüfsubstanz bereits durchgeführt? Wenn ja, mit welchem Ergebnis?** Nicht zutreffend
2. **Angaben darüber, dass in mündlicher und schriftlicher Form aufgeklärt wird. Eine schriftli­che Probanden/Patienteninformation ist beizufügen, in der in allgemein ver­ständlicher Form über Wesen, Ablauf und Ziel des Forschungsvorhabens, studienbe­dingte Änderungen einer Vormedikation, studienbedingte diagnostische Maßnahmen und Belastungstests, Placebo-Gabe während einer Auswasch- und/oder Behand­lungsphase, Therapie in der Vergleichsgruppe und Standardtherapie, Randomisierung, erwarteten Nutzen (z.B. persönlicher Nutzen vorhanden, wenn ja welcher), Komplika­ti­onen, unerwünschte Wirkungen, Dauer der Studie, Fortführung der Therapie am Stu­dienende, Blutentnahmen mit Angabe der Menge, Versicherungsanschrift, Policen­nummer, Telefonnummer, Höchstsumme und die wichtigsten Versicherungsobliegen­heiten informiert wird.**Siehe Probanden-/Patienteninformation
3. **Einwilligungserklärung**

Siehe Probanden-/Patienteninformation

1. **In der schriftlichen Einwilligungserklärung muss auf die Proban­den/Patienteninforma­tion verwiesen werden. Es müssen die im Vorwort aufgeführten Inhalte bestätigt wer­den.**

Siehe Anlage

1. **Angaben darüber, ob Patienten, bei denen Zweifel an der Einsichtsfähigkeit bestehen, in das Forschungsvorhaben aufgenommen werden. In diesem Fall muss ein unabhän­giger Sachverständiger (Facharzt/-ärztin für Psychiatrie), der nicht mit dem For­schungsvorhaben befasst ist, zuvor das Vorhandensein der Einsichtsfähigkeit bestätigt haben.**

Werden nicht eingeschlossen

1. **Angaben darüber, ob Patienten mit eingeschränkter oder nicht vorhandener Einwilli­gungsfähigkeit (z.B. akute schwerwiegende Erkrankung; Bewusstseinseinschränkung; Bewusstlosigkeit; Demenzerkrankungen; Patienten, die unter gesetzlicher Betreuung stehen) in das Forschungsvorhaben aufgenommen werden sollen. In diesem Fall muss eine ausführliche Begründung erfolgen, in der dargelegt wird, dass im Sinne ei­ner sog. Ausnahmeregelung die Bedingungen eingehalten werden, die durch die Stel­lung­nahme der Zentralen Ethik-Kommission bei der BÄK sowie in der europäischen Bio­ethik-Konvention festgelegt sind. Die vorgesehene Forschung muss dem unmittel­baren Nutzen für den Patienten (individueller Nutzen) dienen. Liegt bei gesetzlich be­treuten Patienten eine Grundeinsichtsfähigkeit vor, muss neben der Zustimmung des Betreuers auch der Patient seine Einwilligung erklären**.

Werden nicht eingeschlossen

1. **Angaben darüber,**
2. **ob ein Proband-/Patientenausweis ausgestellt wird**  Nein
3. **ob der weiterbehandelnde Arzt (in der Regel der Hausarzt) über die Teilnahme seines Patienten an dem Forschungsvorhaben informiert wird** Ja, auf Wunsch des Patienten.
4. **dass der Prüfarzt über die notwendige fachliche und persönliche Qualifikation, Erfahrung, Personal, Zeit und Einrichtung verfügt, um das Forschungsvorhaben in der beantragten Weise durchzuführen (curriculum vitae)**

Studienleiterin und alle beteiligten Ärzte sind Studien-/Prüfärzte (Kopie anbei) mit Erfahrung in der Durchführung von klinischen Studien.

1. **dass der Leiter der Einrichtung, an der das Forschungsvorhaben realisiert werden soll, mit der Druchführung einverstanden ist**

Siehe Einverständniserklärung des Klinikdirektors.

1. Kyle RA, Therneau TM, Rajkumar SV, et al.: Prevalence of monoclonal gammopathy of undetermined significance. N Engl J Med. 2006;354:1362-1369 [↑](#footnote-ref-1)
2. Kortüm M. et al.: DGHO-Leitlinien „Monoklonale Gammopathie unklarer Signifikanz (MGUS). DGHO- Leitlinien [↑](#footnote-ref-2)
3. Kyle RA, Therneau TM, Rajkumar SV et al.: A long-term study of prognosis in monoclonal gammopathy of undetermined significance. N Engl J Med 2002;346:564-569 [↑](#footnote-ref-3)
4. http://www.klichi.uni-muenster.de/examate/Schlueter_Monoklonale_Gammopathie_Textskript [↑](#footnote-ref-4)
5. Balderman SR, Lichtman MA. Unusual Manifestations of Monoclonal Gammopathy: I. Ocular Disease. Rambam Maimonides Med J 2015;V6,I3,e0026:1-11 [↑](#footnote-ref-5)
6. Bürki E.: Ein seltener Fall von kristalliner Hornhaut Degeneration. Ophthalmologica 1953;129:211-17 [↑](#footnote-ref-6)
7. Lisch W., Wasielica-Poslednik J., Kivelä T. et al.: The hematological definition of monoclonal gammopathy of undetermined significance in relation to paraproteinemic keratopathy. [↑](#footnote-ref-7)
