# Supplementary figures and images for: Prevalence of corneal findings and their interrelation with hematological findings in monoclonal gammopathy
Source: PLoS One. 2022 Oct 31;17(10):e0276048. doi: 10.1371/journal.pone.0276048 (PMC9621422; doi:10.1371/journal.pone.0276048)

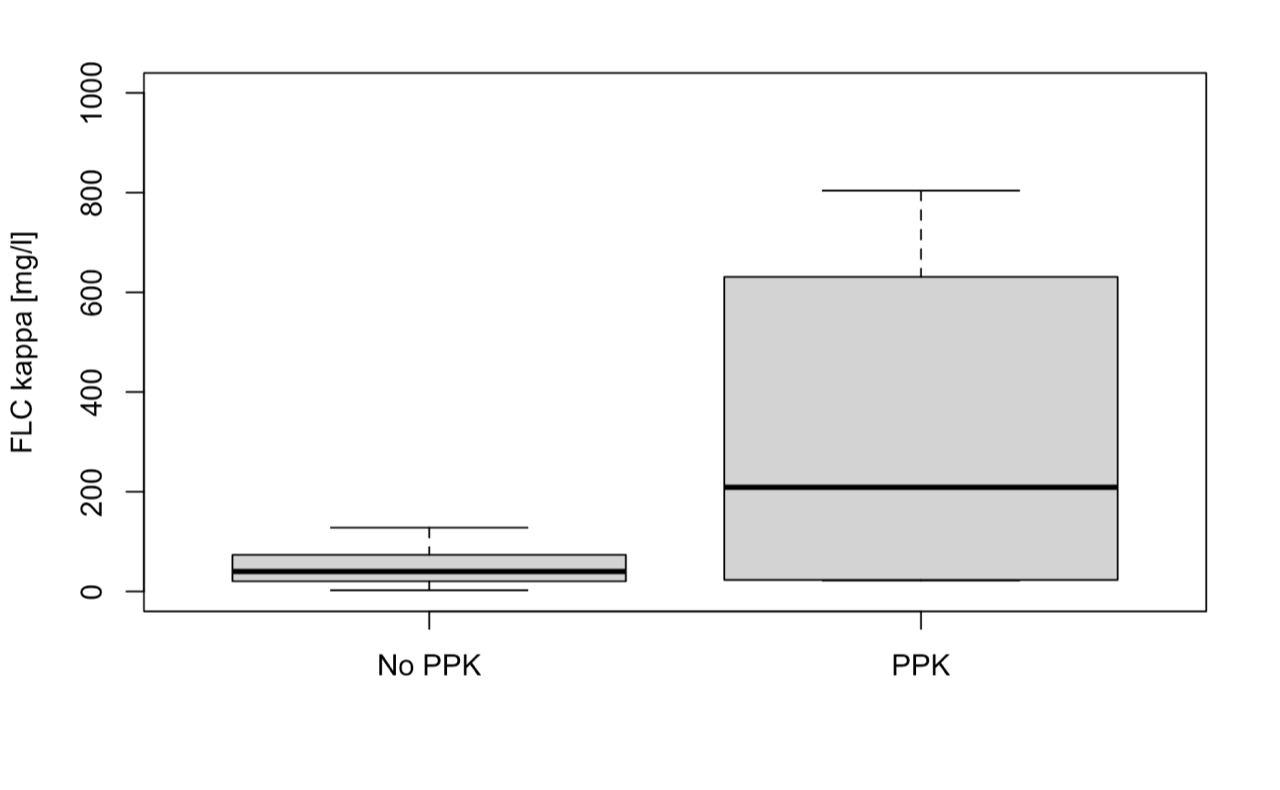

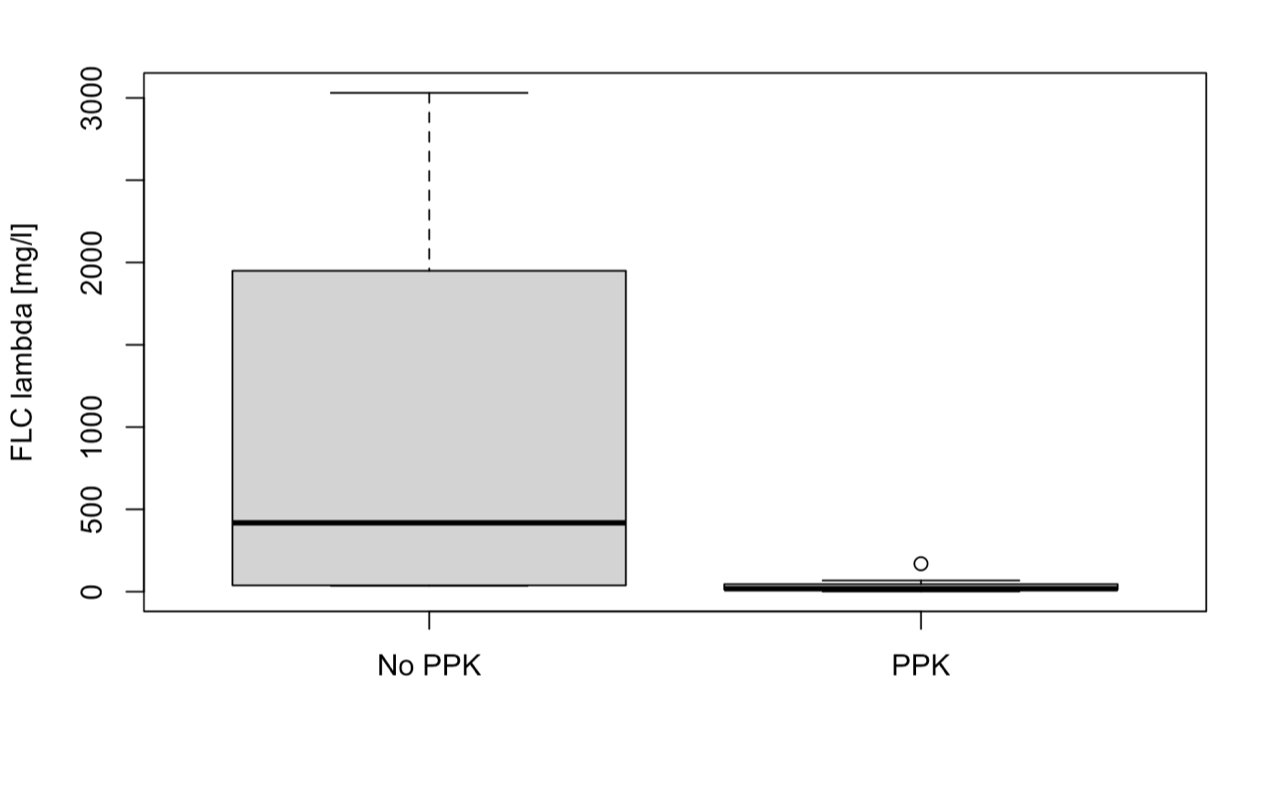

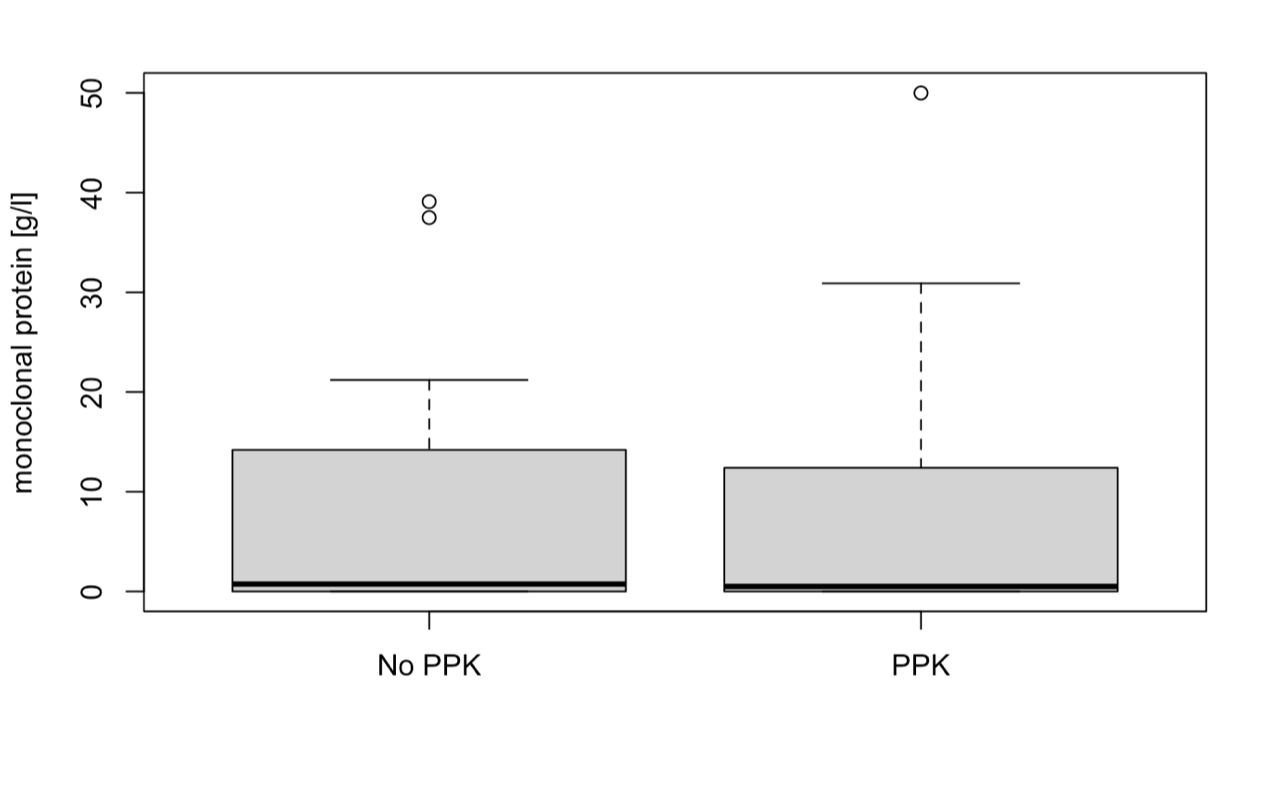

Supplement: S3 File — (DOCX) [file pone.0276048.s006.docx]
