# Supplementary material for: Prevalence of corneal findings and their interrelation with hematological findings in monoclonal gammopathy
Source: PLoS One. 2022 Oct 31;17(10):e0276048. doi: 10.1371/journal.pone.0276048 (PMC9621422; doi:10.1371/journal.pone.0276048)
Supplement: Attachment — Submitted filename: 2021-09-03 Review of PONE.docx [file pone.0276048.s008.docx]

Review of PONE-D-21-09281

The authors report the result of a prospective observational cohort of 51 patients with MGUS, SMM, or MM describing bilateral corneal opacities in an unexpectedly high percentage of patients. Additionally, they describe the clinical courses and managements of two patients with severe ocular affections. The manuscript is well-written, concise and summarizes the findings in a valid manner.

However, before considering publication in PLOSone, I suggest some revisions to the manuscript.

1. Throughout the manuscript, the 51 included patients are divided into patients with a prior hematologic diagnosis (i.e. 45 patients, “group B”) and without such a diagnosis (6 patients, “group A”). One may assume that information on the hematologic diagnosis of these patients has become available at a later point. Please clarify why the distinction between these two patient groups is necessary and provide information on their later hematologic diagnosis or remove the separation into two groups from the manuscript since it is not represented in the figures nor sufficiently discussed.
2. In line 62-63, the “latest classification of PPK” is mentioned and cited. Please expand to provide more information on that classification to the reader.
3. In the introduction, it should be mentioned that MG is not always caused by an underlying plasmacell disorder but can be related to any form of b-cell proliferative disease (Lymphome, CLL, etc.). This is particularly important since the patient case presented later in the manuscript features a lymphoplasmocytoid immunocytoma.
4. Lymphoplasmocytoid immunocytoma should be changed to lymphoplasmacytic lymphoma.
5. Line 75, reference 9 is incorrect and should be changed to [ https://doi.org/10.1159/000068251 ]
6. In line 84, current hematologic guidelines should be cited in addition to reference 17
7. Line 89, correct to “A further goal..”
8. Line 102-103, it is inconclusive why 6 patients without a hematologic diagnosis solely based on the finding of PKK were included in the study considering the given inclusion criteria. Please clarify.
9. Line 103, “MG” is supposed to be “MM”? Please clarify the difference between MG and MGUS as used in the manuscript and make its use in the manuscript consistent.
10. The exclusion criterium of “non-measurable M-protein” appears detrimental to the study because it prevents patients to be included who have had a complete response to therapy or b-cell clones with unmeasurable yet harmful paraproteins (as is the case in various renal paraprotein deposition diseases). Please specify if patients were excluded based on previous therapies, which assays were used to quantify m-protein, and the rational for this exclusion criterium.
11. Line 109, slimCRAB criteria should be used to define MGUS.
12. The concept of “monoclonal gammopathy of renal significance” and “monoclonal gammopathy of clinical significance” should be briefly discussed in this paragraph.
13. Line 116, “special biomarkers of malignancy” should be specified. I suppose this refers to slimCRAB. Also, cast nephropathy is a myeloma-defining event and should be added.
14. Lines 120-130, please make capitalization of parameters consistent.
15. Line 129, “immune fixation” should be “immunofixation”.
16. Line 148, throughout the text, the correct use of “free light-chain” should be observed.
17. Line 150, information on software lacks vendor and country.
18. Flow chart, it seems peculiar that 51 consecutive patients could be included without a single patient not meeting the inclusion criteria indicating screening failure. It appears that patients were included based on their referral from the Department of Hematology, and that screening took place before that. This should be clarified in the text and, if applicable, added to the flow chart. At this point, it is unclear how patients were included in the study given these facts and the fact that 6 patients were diagnosed as primary PPK-patients without preceding hematologic diagnosis.
19. The flow chart is lacking N numbers in some lines (i.e. “0”?). Please correct.
20. A tabular view of the patients’ baseline characteristics should be given as an additional table.
21. A tabular view of the patients diagnoses and ophthalmologic findings should be given as an additional table (e.g. x-axis hematologic diagnosis, y-axis ophthalmologic diagnosis) to present data described in the results section in a more accessible way. This should replace the rather confusion figure 3. Moreover, summarizing patients according to kappa or lambda-paraprotein expression may help extract more information from this cohort.
22. The resolution of the images in figure 2-5 appears rather low. At this quality I cannot assess whether descriptions and arrows are accurate. Resolution should be increased for final publication.
23. Figure 5 d – it should be clarified when the in vivo confocal scanning microscopy was done before or after penetrating keratoplasty and whether this represents an ex vivo specimen or was done before the penetrating keratoplasty. In this case, I suggest rearranging the panels to reflect the fact that panel d predates panel c in time.
24. Do the authors have any information on the subclass of immunoglobulin types? This would provide valuable information and could explain the lack of correlation between MG-type and m-protein levels and ocular affection in this study. The current understanding is that paraprotein depositions are mainly driven by biochemical properties and less by total MG-levels (e.g. proliferative glomerulonephritis with monoclonal immunoglobulin deposits – PGNMID shows a clear correlation to IgG3-kappa subtype). These concepts should be discussed in the manuscript and information on the subclass of Ig should be presented if available.
25. Line 258, the authors should discuss whether MGOS with rapid deterioration of visual acuity (as described in the case vignette) generally warrants systemic cytotoxic therapy.
26. Lines 288-292, as mentioned above, the biochemical properties play an important role in the likeliness of paraprotein to form organized or non-organized deposits and the predilection site of deposition. The authors should expand on the biochemical properties and (mainly size-) differences kappa- and lambda-light-chains and whether size and charge may affect diffusion properties through ocular structures and predilection sites of deposition in the eye.
27. Line 299, in the statement about the statistical significance, the small sample size and rarity of the disease should be discussed as the main obstacle to meaningful statistical analysis rather than speculations.
28. Line 312, “advancement” should be changed to “progress”.
29. Line 313, the concept of “dangerous small B-cell clones” (<https://doi.org/10.1182/blood-2006-03-001164>) should be discussed in this context.
30. Line 335-336, the statement on multicenter trials is rather bold. I suggest recommending setting up patient registries which appears more practical for rare disease such as PKK.
31. Lines 337-338. The sentence (In conclusion,…) needs to be revised to meeting standard English criteria.
32. I encourage the authors to propose an ophthalmologic guidance for the work-up of patients with paraproteinemia: should all patients undergo ophthalmologic evaluation? If not, who should be screened and how often? Does every ocular involvement warrant a cytotoxic therapy? Screening for recurrence after cornea transplantation? Should every patient be transplanted?
33. Line 340-341, the statement that morphology and severity of PPK do not correlate with the stage of monoclonal gammopathy appears bold in view of the given data and should be toned done. Moreover, there are no official stages of monoclonal gammopathy, this term seems misleading and should be corrected.
