# Supplementary material for: Prevalence of corneal findings and their interrelation with hematological findings in monoclonal gammopathy
Source: PLoS One. 2022 Oct 31;17(10):e0276048. doi: 10.1371/journal.pone.0276048 (PMC9621422; doi:10.1371/journal.pone.0276048)
Supplement: Attachment — Submitted filename: Response to Reviewers_PONE-D-21-09281_R2_FINAL.docx [file pone.0276048.s010.docx]

**PONE-D-21-09281**

**Prevalence of corneal findings and their interrelation with hematological findings in monoclonal gammopathy**

**Dear Editor,**

**we thank you and the reviewers for the valuable comments and suggestions and hope to have improved our manuscript now. Below you will find our responses to the editor´s and reviewers´ comments.**

**With kindest regards**

**Mohammad Al Hariri**

**Joanna Wasielica-Poslednik**

Reviewers' comments:

Reviewer's Responses to Questions

**Comments to the Author**

1. If the authors have adequately addressed your comments raised in a previous round of review and you feel that this manuscript is now acceptable for publication, you may indicate that here to bypass the “Comments to the Author” section, enter your conflict of interest statement in the “Confidential to Editor” section, and submit your "Accept" recommendation.

Reviewer #1: All comments have been addressed

Reviewer #2: All comments have been addressed

Reviewer #4: (No Response)

Our answer: thank you for your interest

2. Is the manuscript technically sound, and do the data support the conclusions?

The manuscript must describe a technically sound piece of scientific research with data that supports the conclusions. Experiments must have been conducted rigorously, with appropriate controls, replication, and sample sizes. The conclusions must be drawn appropriately based on the data presented.

Reviewer #1: (No Response)

Reviewer #2: Yes

Reviewer #4: Partly

Our answer: thank you for your evaluation

3. Has the statistical analysis been performed appropriately and rigorously?

Reviewer #1: (No Response)

Reviewer #2: Yes

Reviewer #4: No

Our answer: thank you for your evaluation

4. Have the authors made all data underlying the findings in their manuscript fully available?

The [PLOS Data policy](http://www.plosone.org/static/policies.action#sharing) requires authors to make all data underlying the findings described in their manuscript fully available without restriction, with rare exception (please refer to the Data Availability Statement in the manuscript PDF file). The data should be provided as part of the manuscript or its supporting information, or deposited to a public repository. For example, in addition to summary statistics, the data points behind means, medians and variance measures should be available. If there are restrictions on publicly sharing data—e.g. participant privacy or use of data from a third party—those must be specified.

Reviewer #1: (No Response)

Reviewer #2: Yes

Reviewer #4: (No Response)

5. Is the manuscript presented in an intelligible fashion and written in standard English?

PLOS ONE does not copyedit accepted manuscripts, so the language in submitted articles must be clear, correct, and unambiguous. Any typographical or grammatical errors should be corrected at revision, so please note any specific errors here.

Reviewer #1: (No Response)

Reviewer #2: Yes

Reviewer #4: (No Response)

6. Review Comments to the Author

Please use the space provided to explain your answers to the questions above. You may also include additional comments for the author, including concerns about dual publication, research ethics, or publication ethics. (Please upload your review as an attachment if it exceeds 20,000 characters)

Reviewer #1: (No Response)

Reviewer #2: Authors have made sufficient amendments to the manuscript. Table 1 should provide summarizing data of all patients and not only subgroups.

Our answer: thank you for your recommendation. We added summarizing data of all patients.

Reviewer #4: The authors revised their manuscript and addressed some of the concerns posed by the reviewers. However, the manuscript still needs extensive revisions. Even though the language and grammar are mostly ok, the manuscript is not well organized. They may benefit from a professional editing service to better construct the manuscript.

Our answer: thank you for your comment. We strive to improve our manuscript and thank you for your suggestions for improvement.

Overall comments:
1. This study is not a clinical trial. If it were, there would be an intervention investigated. What was the intervention investigated here? This is a prospective observational cross-sectional study. Therefore, its category should be changed, and then CONSORT will be irrelevant and not mandated by the journal.

Our answer: thank you for your comment. In fact, our study is an observational cross-sectional study without intervention. We changed this category in the methods section and removed the CONSORT flow diagram as recommended.

2. Authors should be very clear and consistent about what their hypothesis was, what primary and secondary aims they had to conduct this study, and what main outcomes were selected. At the end of the introduction, the authors state, “The aim of the present study was to investigate whether the type and severity of the MG determines the presence, morphology, and severity of the PPK.” They should stick with this throughout the text, and their methods and results should reflect this. Their rebuttal letter says that the primary aim was to find out the prevalence. Which one? Their main outcome(s) should be determined based on that.

Our answer: thank you for this comment. We agree that the aims and hypotheses of the study were not clearly defined in the previous versions of the manuscript. Our primary aim was to determine the prevalence of PPK in the MG population. Our secondary aim was to investigate whether the type and severity of the MG determines the presence, morphology, and severity of the PPK.

Therefore, we changed the title; reformulated the purpose in the abstract; defined the aims and hypotheses in the last paragraph of the introduction; added titles of the subsections in the results section; and discuss our results according to the aims and hypotheses in the first two paragraphs of the discussion.

3. Study groups are usually formed to compare two or more groups regarding main outcomes to address the primary and secondary aims. The way the authors presented groups A and B is not scientifically acceptable. Participants included in each group represent two different cohorts. The authors chose to include group A into the analysis when they see the fit and exclude, which is a critical flaw. Participants in group A and group B cannot be expected to make a cohort as they were enrolled based on different characteristics. The statistical analysis would be biased if some patients were screened due to having a hematologic diagnosis and found to have a corneal disease, and some were seen in ophthalmology clinics maybe due to visual symptoms or diagnosed incidentally. These two groups cannot be merged for some part of the analysis conducted here. They should exclude those additional 6 patients and rearrange the whole manuscript accordingly. Their justification for including those 6 patients and then excluding them from the analysis is not scientifically convincing.

Our answer: thank you for this comment. We created groups A and B for better understanding of the origin of our patients. Group A consisted of patients with the primary ophthalmological suspicion of PPK (and a confirmed diagnosis of MG) and group B consisted of patients with the primary hematological diagnosis of MG.

We agree that the groups were not acceptable. Therefore, we deleted “groups A and B” throughout the manuscript. We modified the Methods section (lines 124-133) and highlighted that the prevalence was evaluated only in patients with the primary hematological diagnosis (Methods lines 127-128). In the Results subsection “Prevalence of PPK”, we added table 2 considering only 45 patients recruited in the Department of Hematology.

However, the patients recruited at the Department of Ophthalmology (primary PPK suspicion) fulfill the in- and exclusion criteria of the study as they were diagnosed with MG and provide many valuable information regarding the secondary aim of the study (interrelation between corneal and hematological findings). Therefore, we don´t want to exclude them from this part of the study and to lose valuable information on this rare disease. We added this information in the Methods section (lines 129-133) and in the Results section (lines 217-220).

4. This study is conducted at one-time point. Cross-sectional. It was not a cohort study with long-term follow-up. They did not mention any follow-up in their methods. They did not mention any treatment (surgical or systemic) in their methods as a study intervention either. They cannot select two patients and talk about their surgical treatments and results. They also cannot report the results of the systemic treatment in one of them. The authors squeezed two case reports at the end of the results, which is not right. The study design should have been different if they were investigating the efficacy and safety of a surgical intervention (PK) and/or systemic treatment. They stated in their rebuttal letter that they are preparing another publication to report follow-up data. This would belong to that manuscript. They should remove it from this manuscript.

Our answer: thank you for recommendation. We removed this part from the manuscript.

5. In their rebuttal letter, some of the points were not addressed; however, they responded as if they had made changes. For instance, they said they added n for MGUS, SMM, and MM in the abstract, but it was not added. Another table including demographic and clinical characteristics according to PPK status was suggested, and they said it was added, but there is no such table.

Our answer: we sincerely apologize for these unintentional mistakes. This time, we surely added n for MGUS, SMM and MM in the abstract. We added table 1 with demographic and clinical characteristics according to PPK of all patients in the Results section.

Specific comments:
Line 113-114. “primary diagnosis of PPK without any known hematologic disorder” - PPK diagnosis cannot be done without known hematologic disorder and/or cornea biopsy.

Our answer: thank you for this comment. We change it to primary suspicion.

Lines 174-178. More details are needed re: histological evaluation. What tissue samples did they mean here? How were they collected?

Our answer: “Histological evaluation” was part of description of the surgical cases. The whole part was now removed on your request.

Lines 180 to 191: Was a normality test performed? Which one? What were the main outcomes?

Our answer: we did not perform a normality test, but we checked the distribution in the histogram. The data in the histogram were not normally distributed.

Line 197: Were both eyes included in the analysis? Please clarify. Data obtained from the right and left eyes are correlated and not recommended to be included in the analysis. Either a factor analysis would be necessary to account for this effect or another appropriate analysis method. Most commonly, only one eye would be included in the analysis. Authors are expected to clarify this in the methods section.

Our answer: Both eyes were included in the analyses. Ocular findings were the same in both eyes in all patients. In the analysis of ocular involvement in MG, either both eyes were involved or not. Thus, we decided to include the mean value of right and left eyes into the analysis, which is a common procedure if always both eyes are affected in an individual subject. We have included this in the methods section (lines 184-187). In the analysis of visual acuity, corneal thickness, and densitometry, involved vs. uninvolved eyes were examined.

Table 1. MGUS, SMM, and MM should make the columns. Units of measurements should be added in relevant areas. Should include whether those results within the age columns are mean and SD or something else. As described in the statistics section, they should report the frequency of the categorical values.

Our answer: As you previously wished we rearranged table 1 to be according to PPK status, made MGUS, SMM and MM to columns and included the information of age frequency of the categorical values in the table.

Line 247 and 257. Recommend presenting in a table. This was suggested before, and the authors responded as if they did.

Our answer: we apologize for this misunderstanding. The requested table is now provided.

Lines 271 to 290. This paragraph does not belong to this paper as suggested above. This is not a longitudinal study, no follow up was planned for this study. Should exclude from this paper and include in the follow-up paper they mentioned. If they want to mention the necessity of PK in two patients, they can include a variable/parameter as PK recommended yes/no and discuss it but cannot include PK results during the follow up and discuss.

Our answer: Removed

Lines 314-330: Can discuss that two patients had a significant corneal disease and were recommended PK but cannot discuss long-term treatments, systemic treatments given to these patients as cannot be reported as part of this study.

Our answer: Removed

336-340: “This is probably due to the fact that the “non-crystalline” PPK types were unknown and subsequently overlooked or misdiagnosed. The most common form of PPK in the present study is a discreet stromal haze, which can easily be overlooked. The prevalence of crystalline keratopathy in our study was 2% (1 of 45 patients) and, hence, comparable with the prevalence found by Bourne at al.” and the following paragraph are essential points of this study.

Our answer: thank you! We do think that PPK is mostly a very discreet finding – “crystals” may be seen in the confocal microscopy, but not on a slit-lamp. We would like to work on it the next paper..

Lines 373-381: They mention in statistical methods that they did the Spearman correlation test. No correlation results were reported in the result section. Then, they discuss here some results. They use different terms almost interchangeably, such as no association, statistically significant risk factor, and correlation, and report some p values. Were these reported in results? What statistical methods revealed these results exactly?

Our answer: Thank you for this correction. Initially, we planned to perform Spearman correlation, but as the PPK status is a dichotomous variable, Mann-Whitney-U testing showed the same information indicating no association between the level of paraprotein and disease status.

We removed Spearman correlation from the “statistical analysis” and modified some parts of the discussion (please see lines 470-502).

Lines 441-443: “We consider systemic therapy necessary in case of MGOS, especially before the penetrating keratoplasty. Nevertheless, a lifelong follow-up after corneal transplantation is necessary to rule out a corneal recurrence.” Not the result of this study, cannot conclude that based on this study findings. Should consider removing.

Our answer: We removed this paragraph.

7. PLOS authors have the option to publish the peer review history of their article ([what does this mean?](https://journals.plos.org/plosone/s/editorial-and-peer-review-process#loc-peer-review-history)). If published, this will include your full peer review and any attached files.


If you choose “no”, your identity will remain anonymous but your review may still be made public.


**Do you want your identity to be public for this peer review?** For information about this choice, including consent withdrawal, please see our [Privacy Policy](https://www.plos.org/privacy-policy).

Reviewer #1: No

Reviewer #2: **Yes:** Linus Voelker

Reviewer #4: No

[NOTE: If reviewer comments were submitted as an attachment file, they will be attached to this email and accessible via the submission site. Please log into your account, locate the manuscript record, and check for the action link "View Attachments". If this link does not appear, there are no attachment files.]

While revising your submission, please upload your figure files to the Preflight Analysis and Conversion Engine (PACE) digital diagnostic tool, <https://pacev2.apexcovantage.com/>. . PACE helps ensure that figures meet PLOS requirements. To use PACE, you must first register as a user. Registration is free. Then, login and navigate to the UPLOAD tab, where you will find detailed instructions on how to use the tool. If you encounter any issues or have any questions when using PACE, please email PLOS at [figures@plos.org](mailto:figures@plos.org). Please note that Supporting Information files do not need this step.

*In compliance with data protection regulations, you may request that we remove your personal registration details at any time.* [*(Remove my information/details)*](https://www.editorialmanager.com/pone/login.asp?a=r)*. Please contact the publication office if you have any questions.*
