# Supplementary material for: Prevalence of corneal findings and their interrelation with hematological findings in monoclonal gammopathy
Source: PLoS One. 2022 Oct 31;17(10):e0276048. doi: 10.1371/journal.pone.0276048 (PMC9621422; doi:10.1371/journal.pone.0276048)
Supplement: Attachment — Submitted filename: Response to Reviewers_PONE-D-21-09281_R3_25.09.22_MA.docx [file pone.0276048.s011.docx]

Mainz, 27.09.22

PONE-D-21-09281_R3

**Prevalence of corneal findings and their interrelation with hematological findings in monoclonal gammopathy**

Dear Editor,

we thank you and the reviewers for the valuable comments and suggestions and hope to have improved our manuscript now. Below you will find our responses to the reviewers´ comments.

With kindest regards

Mohammad Al Hariri

Joanna Wasielica-Poslednik

**Reviewer #5: The aim of the study was to determine prevalence of paraproteinemic keratopathy among patients with monoclonal gammopathy, and to evaluate possible interrelation between corneal and hematological parameters.**

**The findings are of interest, and they are put in the context of previous publications. The authors have addressed the comments raised in the previous round of review.**

**Still, there are couple of points that should be corrected or described more clearly:**

**Abstract:**

**r.40-41 The conclusion that „Median level of free light-chain 40 (FLC) kappa in serum of patients with kappa-restricted plasma cell dyscrasia was higher in 41 patients with PPK compared to patients without PPK (p=0.18)“ is not correct because the p value does not reach the level of significance**

Thank you for this comment. We changed this part of the abstract (lines 38-42).

**Methods and Results:**

**The „PPK prevalence and morphology“ is described first in the“ Results section, then the „Hematological findings“, and finally the „Ophthalmological findings“. However, The PPK prevalence and morphology belongs to the Ophthalmological findings, too. The results should be presented more systematically.**

Thank you for this suggestion. We changed the name „ocular findings“ to „clinical findings“ and swapped the order of the last two parts of the results. We hope to have improved the understanding.

**Was the IVCM examination performed in patients with diagnosis of PPK, only? In the view the densitometry results were not different between the patients with and without PPK, and the fact, the previous studies have found a difference in densitometry between the MG patients and age-adjusted controls, it could be possible that some pathological findings could be proven even in the „non-PPK“ patients using IVCM! This should also be mentioned in the discussion along the fact the control group is missing.**

IVCM examination was performed in all patients as it was a prospective study – we added this information in the first sentence of the „Ophthalmological evaluation“ section (line 166).

We found hyperreflective needle-like crystalline stromal deposits using IVCM in all corneas with the diagnosis of PPK on the slit lamp examination (Results section/IVCM lines 250-251).

In addition, 18 other patients had less pronounced hyperreflective point- and/or needle-like deposits in the deep stroma without any discernible opacities in the morphological examination of the cornea using the slit lamp. We added this information in the Results section/IVCM (lines 252-254).

We added the following sentence in the discussion (lines 378-381): “Furthermore, using IVCM we observed hyperreflective point- and/or needle-like deposits in the deep stroma of patients without any PPK-like opacities visible on slit lamp. Future clinical trial with a healthy control group should answer the question if IVCM could detect the “pre-slit-lamp” PPK forms.”

We agree that the limitation of the study is a lack of a control group. We mention it in the discussion (line 466). We plan to include a control group into further studies on this topic.

**In the Methods section, there is indicated that tonometry, fundoscopy, and macular OCT was also performed to rule out the other ocular involvement , but no outputs are mentioned in the Results section.**

Thank you for this advice. We added this information in the „Clinical Findings“ section.

One patient with Waldenstrom's disease had bilateral macular involvement in form of a subfoveal defect of the retinal pigment epithelium with cystoid macular edema and one patient with SMM type IgA λ had macular involvement in the form of a focal detachment of the neurosensory retina with secondary choroidal neovascularization (CNV). We interpret these findings as paraproteinemic maculopathy. We added these information in Clinical findings (lines 268-275).

**Discussion:**

**r.331-332 The prevalence of crystalline keratopathy is presented. However, this is not described in the „Results section“ earlier. Also, check the form „crystalline“ is used all over the text, not „cristalline“ (e.g. r.336 „non-cristalline“)**

Thank you for the remark. We added this information in the Methods section (lines 218-221).

We use term "crystalline" within the manuscript.
